# Exploring Participation and Interaction in a Bottom-Up Health Promotion Program for Migrant Women in Norway

**DOI:** 10.1177/1049732320980397

**Published:** 2020-12-21

**Authors:** Yan Zhao, Trude Gjernes, Marianne Hedlund

**Affiliations:** 1 Nord University, Bodo, Norway; 2 NTNU, Trondheim, Norway

**Keywords:** migrant women, health promotions, health education, “health parties”, interaction, trust, cultural health capital, Norway, health innovation, participant role, participant observations, semi-structured interviews

## Abstract

In this article, we examine the participation and interactions of migrant women and experts who attended health parties. Based on data from participation observations and semi-structured interviews from participants of health parties, we examine how health parties may be considered an innovative bottom-up community program that could influence how migrant women focus, learn, and discuss health issues as well as interact with health care. Through a qualitative analysis, the article demonstrates two ways of organizing health parties and different approaches to the health expert role, and how this impacts the social setting and interactions of a health party. In the Norwegian setting, migrant women are considered by health authorities to be the most difficult group to reach due to cultural and language barriers. Health parties may represent an alternative for bridging these barriers and may create a useful context for active participation and learning about health for migrant women.

In this article, we examine participation and interaction facilitated by a bottom-up health promotion program for migrant women. The program, which features “health parties,” is unique because it was developed by a voluntary organization established by migrant women in Northern Norway, Kvinnenettverket Noor (hereafter NOOR). Inspired by the concept of the Tupperware party, health parties are events in which the host invites female friends, family, and acquaintances to a “party” with the goal of not only socializing but also sharing information about health and the health care system in Norway. Depending on the chosen topic of the health party, one or two health experts are invited to provide information about a specific health issue. These experts are usually health professionals with particular specializations, from diabetes nurses, gynecologists, and midwives to nutritionists and therapists. The program uses parties as a context for discussing health issues and sharing experiences and thus creates a space for transforming the expert knowledge of professionals into common knowledge among participants. From September 2015 to March 2016, the NOOR cooperated with several local migrant organizations/communities and coordinated 11 health parties, which covered topics such as asthma and allergies, diabetes and diet, reproductive health, mental health, and thyroid disorders.

Based on observations and qualitative interviews, this article examines the participation and interactions of the migrant women and experts who attended the health parties. Our investigation focuses on how different ways of organizing health parties shape frameworks for discussing and sharing relevant knowledge about issues related to health and health care. It also examines how different forms of participation and interaction necessary for realization of such discussions were facilitated. In addition, we explore which participant roles and social relations were enacted in the health parties among experts, hosts, and migrant women. By addressing these issues, this article suggests innovative approaches to modeling health information for migrant women.

With some exceptions, there have been few ethnic minorities in Norway (Eriksen, 2013) and Norwegian society was considered rather homogeneous until the late 1960s (Brochmann, 2003). However, as in many other European countries, the migrant population of Norway has increased continuously in recent years: In January 2020, the total migrant population was 979,254, constituting 18.2% of Norway’s total population. Of this figure, 577,847 people were migrants from the Global South (Statistics Norway, 2020). Research shows that migrants, particularly those from the Global South, and those who are women, have generally poorer health outcomes than the general population in Norway (Attanapola, 2013; Diaz et al., 2015; Forland, 2009; Norwegian Ministry of Health and Care Services, 2013). Migrant women are more likely to be exposed to certain illnesses and health problems such as diabetes, HIV, hepatitis B, tuberculosis, vitamin D deficiency, and mental health problems (Spilker et al., 2009). They may also face a higher risk of hyperemesis as well as anemia, diabetes, pelvic girdle pain, and other pathological diseases (Austveg, 2010; Jenum, 2010).

In a welfare state like Norway, the health care system is funded primarily by the government and aims to provide universal coverage based on the principle of equality. However, studies indicate that some migrant groups from low-income countries have reduced access to health services due to economic, organizational, language, and cultural barriers (Diaz et al., 2015; Norwegian Medical Association, 2008; Norwegian Ministry of Health and Care Services, 2013). Studies in both Norway and other Organisation for Economic Co-operation and Development (OECD) countries have discovered that migrant women’s premigration experiences and cultural perceptions of health and illness such as menstruation, pain, pregnancy, and childbirth can further contribute to barriers to accessing health care and achieving safe medical practice in host countries, leading to negative health outcomes (Austveg, 2010; Hawkey et al., 2016; Henry et al., 2020). Facing a complex health care system in which individuals are expected to take ever more responsibility for their own health (Finbråten & Pettersen, 2012), migrant women in Norway can find it challenging to access the appropriate service for their needs.

It is in this context that the NOOR launched the health parties project. NOOR’s aim in developing the project was to promote migrant women’s health by increasing their awareness and knowledge about health and the health care system. The intention of the project was that the health party should be a method to improve user-controlled and customized health information for migrant women. The health party project was not guided per se by empowerment and dialog ideology, in the sense that these ideologies were not preannounced for migrant women or health experts attending the health parties. Rather, the health party should be an arrangement to learn and share knowledge about health issues, common diseases, and how to address the health and social care system.

In this article, we do not determine whether the health party project was successful in the long run. Rather, the article focuses on how different ways of organizing the health parties influence and facilitate the roles and interactions of migrant women and health experts who participate in the parties. Hence, we stress the significance of the interactive dynamics to enhance migrant women’s participation and learning and how organizational frameworks matter for a bottom-up, lay health education program.

Women play a major role in managing health and illness in their families (Clarke & Olesen, 1999). Yet, migrant women, and particularly those who stay at home, comprise the group considered by Norwegian public health authorities to be the most difficult to reach due to cultural and language barriers (Abebe, 2010; The Norwegian Ministry of Health and Care Services, 2013). Thus, whereas ethnic Norwegians tend to have a high level of institutional trust, some migrant groups have little trust in state institutions and rely more upon informal and personal relationships to maintain their ontological security and identity in diaspora (Engebrigtsen, 2007; Hopkins, 2006; Næss, 2019). Studies also show that health professionals who share the same ethnic and cultural background can positively influence the engagement and integration of migrant communities in the health care system (Næss, 2019). For these reasons, relationships and interactions that emerge during the health parties will be of interest.

## Theoretical Perspectives

This article analyzes health parties as providing an alternative arena for acquiring health-related knowledge. Health-related knowledge may be divided into three categories: health promotion, disease prevention, and health service use and navigation (Willis et al., 2016). Places where health-related knowledge is communicated and discussed are considered learning arenas. These may be formal settings, such as general practitioner (GP) offices, hospitals, and public health centers, where public or private health services are provided to people, or informational meetings about health issues, such as mental health, diabetes, cancer, or dementia, that are arranged by health service providers in collaboration with voluntary organizations. There are also more informal arenas where health matters are discussed among people, such as social gatherings, and announced either through traditional modes of media or through social media.

Traditionally, health promotion focuses on health determinants and most health promotion programs adopt a behavioral approach, which focuses mainly on individual lifestyle decisions and addresses recognized health risk behaviors, such as tobacco smoking, high consumption of alcohol, high-fat diets, and lack of exercise (Baum & Fisher, 2014). Several studies (Baum & Fisher, 2014; Gjernes, 2004, 2008, 2010; Petersen & Lupton, 1996; Smith & Anderson, 2018; Williams & Fullagar, 2019) have demonstrated clearly that such behavior-oriented health advice often has a limited effect or that the positive effects are mainly experienced by members of the more privileged social classes (Abel & Fröhlich, 2012; Baum & Fisher, 2014). This kind of health advice is often based on one-way communication, in which a health expert informs the public about how to avoid health risks. However, such advice often ignores exciting and relevant lay perspectives on the matters discussed, or the practical implications of recommendations expected to be internalized and implemented (e.g., a healthy diet), for people’s economic situations and everyday life practices. Hence, individualistic and behavioral health promotions have been criticized for a lack of recognizing the lifeworlds of various social groups, and the experiences and lay health knowledge of the people these programs seek to reach (Abel & Fröhlich, 2012; Baum & Fisher, 2014; Gjernes, 2004). In this context, our study on health parties yields valuable insights into engaging the lay perspectives of ordinary people in health promotion and of specific groups who may not share the lifeworld or cultural context of the social majority. Our analysis also touches on how migrant women relate to *system knowledge*, that is, knowledge about how health care systems are organized and operate, which is necessary to utilize Norwegian health services. Willis et al. (2016) demonstrated the importance of system knowledge for accessing appropriate hospital treatments and the significant involvement of social networks in providing such knowledge.

We have adopted a participation framework (Goffman, 1981) to study health parties as an alternative bottom-up and community-based platform for migrant women to communicate about health and health care topics. According to Goffman (1974, 1981), in social situations and during social interactions, participants may take on various roles during social exchange or they may be assigned these roles by other participants. These roles determined by situations and interactions are dynamic and often unconsciously adopted. Participant roles are relational and often influenced by cultural traditions, institutional rules, or power relations. Participants may experience their roles as assigned by others and thus something over which they have no control or free choice. Moreover, such extrinsically assigned participant roles may influence how participants understand themselves in relation to others, and vice versa. Some participant roles are formally constituted (i.e., expert, patient, audience member), whereas others are situationally, interactionally, and locally established.

## Method

This article is based on data collected from the NOOR’s health party project. Data include participant observations and semi-structured interviews and were collected from seven health parties that took place among different migrant communities and groups in Nordland county, Norway, from September 2015 to March 2016. Six semi-structured interviews were conducted with five hosts of the health parties and the last interview was performed with a health expert who attended several health parties. Table 1 provides an overview of the observed health parties and the interviewees.

**Table 1.** Overview of Observed Health Parties and Interviewees.

Health Party	Host	Place	Topic	Guests (*n*, Background)	Info. Provider
1	Migrant Organization A (Interviewee)	A borrowed meeting room	Asthma and allergies	5, members of the same organization	Representatives of AAF^a^
2	Leader of Organization B (Interviewee)	The host’s home	Thyroid disorders	9, friends of the host	Representative of SF^b^ who happens to be a GP
3	Same as Party 1	Same as Party 1	Diabetes and diet	6, members of the same organization	Representatives of DF^c^ and one nutritionist
4	Migrant Organization C (Interviewee)	A borrowed meeting room	Mental health/coping with stress	16, potential members of the organization (Announced as an open event)	Therapist/ Medical yoga coach
5	Project leader at NAV^d^ (Interviewee)	A meeting room at NAV	Reproductive health	8, participants of the project	Gynecologist (Interviewee)
6	One key figure of Migrant Organization D (Interviewee)	The host’s home	HPV and the screening program	11, friends of the host from the organization	Gynecologist (Interviewee)
7	Another key figure of Migrant Organization D	The host’s home	Menopause	12, the majority of attendees at Party 6	Gynecologist (Interviewee)

*Note.* GP = general practitioner; HPV = human papillomavirus.

a *Asma og allergiforbundet*, the Patient Organization for Asthma and Allergy. ^b^*Stoffeskifteforbundet*, the Patient Organization for Thyroid Disorder. ^c^*Diabetesforbundet*, the Patient Organization for Diabetes. ^d^*Nye arbeids- og velferdsetaten*, Norwegian Labour and Welfare Administration.

Yan Zhao performed the data collection using an ethnographic approach based on participant observation (Atkinson, 2015). Accordingly, the researcher aimed to “enter into the world of others” and “engage” or “interact with them.” The researcher was introduced as a researcher at the beginning of each health party. She interacted socially and joined activities included in these health parties, such as food sessions, group discussions, and quizzes. The researcher has a migrant background herself, which made it easier to gain trust among participants. She observed the activities and conversations at the health parties over a period of 1 to 2 hours and only intervened if explicitly invited by other participants. In order not to disturb participant interactions, no audiovisual recordings of the parties were done. Instead, the researcher made detailed notes during the parties and used her memory to supplement/complete these notes immediately following each party to reduce the transformational problems associated with long-term memory in qualitative research (Zhao, 2017). The notes included descriptions of interactions and transcriptions of parts of relevant discussions. The participants are anonymized in the text.

The five hosts were interviewed following the respective health parties they hosted. All hosts were migrants from Asia, Africa, and Latin America, apart from one person who was a project leader from the Norwegian Labour and Welfare Administration (NAV). The interviews primarily covered how the health parties were organized and prepared, in addition to the hosts’ motivations for hosting the parties, and their experiences and reflections of arranging the parties. A gynecologist with a migrant background attended several parties as a health expert and was interviewed last. The interview with the gynecologist covered her migrant background and how this had contributed to generating trust among participants, her health expertise and knowledge about the Norwegian health care system, and which translation issues might appear for migrant women. All interviews lasted around 1 hour and were recorded and transcribed verbatim.

Ethical approval was granted by the Norwegian Data Protection Service (NSD; NSD Project No. 46809). Anonymity and discretion were secured as a condition of data access. Informed consent was provided by all participants and all interviewees for each health party. The NAV project leader and the gynecologist were also informed about the difficulty of anonymizing their identities due to their specific roles or background in the project and the relatively small context. Even with anonymity, their identities could be disclosed through “back way identification” (Hvinden, 1994). Nonetheless, they both agreed to be interviewed and stated that they participated in the study in their professional, rather than private, capacity. Consequently, they gave their consent with no guarantee of anonymity. In this article, we have sought to anonymize these two interviewees as far as possible, and extra caution has been exercised when presenting data concerning them.

All authors participated in the data analysis. Following Rapley (2011), field notes and interviews were thematically coded after which data were refined and analyzed inductively. We first analyzed the observation notes, examining both the form and content of each health party, with a special focus on participant interactions. The questions we asked included the following: What information was provided by health professionals and in what ways? How did the guests react to this type of information? What questions were asked and discussed? Thereafter, we analyzed the interview data to explore further details about the themes and patterns emerging from the observation notes. This was done to explore data patterns of the interactions observed at the health parties and any links to party organization or provision of data. We went back and forth between observation notes and interview transcripts, (re)interpreting these types of data in relation to each other and as part of the whole.

## Results

The results are organized according to the main patterns found in the analysis. The first pattern we describe is how two ways of organizing health parties set premises for possible interactions and enactment of various roles among participants of the health party. The second pattern described the significance of health experts’ roles for the interactions and sharing of experiences and knowledge among participants.

### Two Ways of Organizing Health Parties

As shown in Table 1, each health party was conducted in a different manner, from the size and location to the composition of guests and topic of discussion. We found that how a health party was organized influenced the interactions, dynamics, and participant roles. Apart from Health Party 5, which was arranged by the NAV project leader, all health parties were hosted by migrant women who were key figures in different local migrant organizations/communities. Some hosts acted as a representative of their organization while others as private individuals. For example, two interviewees emphasized that the health parties they hosted were their own private parties, whereas two interviewees expressed that they hosted the parties on behalf of their migrant organizations. Consequently, we identified two types of health parties: those hosted by an individual as a *private event* (Health Parties 2, 6, and 7) and those hosted by an organization as an *organizational event* (Health Parties 1, 3, 4, and 5).

In organizational health parties, the hosting migrant organization had either invited all female members of the organization (Health Parties 1 and 3) or arranged it as an open event for the entire community that her organization represents (Health Party 4). When NAV arranged Health Party 5, it was incorporated into NAV’s own program for migrant women, which requires obligatory attendance of the female members of the NAV program. These health parties were held at public- or semi-public locations, such as a meeting room belonging to the host organization or a meeting room borrowed from another voluntary organization (e.g., the Red Cross). By contrast, when health parties were arranged as private events, they took place in the hosts’ private homes. Hosts selected their guests according to their personal preferences and invited mostly friends. For example, one interviewee and her group arranged two health parties; the guests were a group of acquaintances who knew each other well privately and met regularly on other occasions.

There was also a difference in the framing of health parties as *parties*. All three private health parties were arranged as a casual dinner party, where all participants, including the health experts and observing researcher, were invited to taste dishes or finger foods prepared by the host. For Health Parties 6 and 7, several guests even helped prepare the food. Conversely, the organizational health parties often started with a presentation on the chosen topic directly after all guests had arrived. While the organizational hosts prepared coffee, tea, fruit, and snacks, only some of the guests helped themselves either before the health parties started or during the break. These two types of health parties represented different social settings, which had implications for the interactions and participation of the guests and their communication with the health expert.

The learning activities at a health party (both private and organizational) consisted of two parts: a presentation by a health expert on the chosen topic, followed by a more interactive period with questions and discussion. The health experts had prepared a PowerPoint (PPT) presentation in all parties except Health Party 1. In private health parties, the PPT was displayed either on a television screen or with a borrowed data projector. The data analysis from both observations and interviews confirmed that participants found the PPT presentation useful. Two hosts said that this improved guests’ understanding because “the Norwegian language has difficult pronunciations” and “many immigrants are better at reading than listening.” This may particularly be the case for specific medical terminology. We also observed that guests often referred to the PPT slides when asking questions. In the presentation section of the learning activities, we found no distinction between private and organizational health parties in terms of the interactions between health experts and guests. In both cases, these consisted predominantly of a monologue by the health expert, punctuated by a few sporadic questions from the guests.

However, there was a clear distinction between the private and organizational health parties regarding active participant roles and interactions in the second part of the health party when migrant women were expected to be active participants. The contrasting level of participation engendered by the different health party types is illustrated by the following quote from an interviewee (host of Health Party 6) who originally planned to host a health party on behalf of her migrant organization, but then changed her mind due to a lack of support within the organization:

Interviewee: I thought afterwards that it was actually good that I chose to have it privately, at my home. It was not a very big group. Those who were there, we knew each other very well. Then they [the guests] became more engaged, felt freer to talk. As you can see, we really enjoyed being together. That evening was just cozy. In addition, we all got something useful.

Interviewer: So, you think it would work less well if it were hosted by your organization?

Interviewee: If it is done in a bigger context, organized by an organization, you just sit there and listen, just like participating in a lecture. Nobody dares to ask [questions], right?

This quote confirms findings from observation data that participants were generally more active in private health parties than in health parties hosted by organizations.

The analyzed observation data showed enactment of different participation roles among the guests. In the organizational parties, participants mostly asked concrete questions of the health expert, primarily to clarify points from the presentation. For example, at Health Party 3 (topic: diabetes and diet), the guests asked, “What does ‘autoimmune’ mean?” “If potato does not belong to the category of vegetables, how about sweet potato and taro?” and “If I do not have high blood sugar, should I also avoid these fruits?” Such questions often required only a short answer from the health expert and led to few discussions among the guests. Still, these concrete questions do indicate an internal process in which the participants attempted to “translate” the systematic information from medical discourse into their everyday context. The observation data also showed there was less initiative among the guests and that it was mostly the health experts who initiated interactions with the guests in the organizational parties. For example, at Health Party 1 (topic: asthma and allergies), the health expert handed out some trial products such as washing powder and shampoo, which were asthma- and allergy-friendly. By doing so, the health expert also taught the guests how to check the ingredients of products and determine whether a product is asthma- and allergy-friendly. At Health Party 3, the health expert gave participants a quiz, asking questions such as, “How much sugar is there in different food products?” To invite more responses from the group, she encouraged guests to discuss with one another and to answer the questions together. This indicates that group participation and interaction also tended to be initiated and driven by the health experts in organizational health parties.

The private health parties were to a larger degree characterized by more active participation from the guests who shared their knowledge and personal experience. At Health Party 6, the topic was human papillomavirus (HPV) and the screening program, and the health expert was the gynecologist. After she had finished her presentation and answered two short questions from the guests, the host commented, “Oh, I didn’t know the participation rate in the screening program was this much lower among migrant women.” Here, she referred to statistics presented by the gynecologist. The host then looked at her guests and said, “I think we would be smarter to go to the doctor and take the test.” This comment precipitated the following discussion, in which several guests participated actively:

Guest A: I’ve had a gynecological examination each time I’ve been back to [home country].

*[Guests B and C nodded, indicating that they did the same.]*

Gynecologist: Why don’t you do it here?

Guest A: I use a private clinic in [home country] and I always go to the same doctor, a female doctor. I feel safe and comfortable being checked by her.

Host: Was it about language?

Guest A: Hmm . . . well, maybe. Because I often asked her a lot of questions each time I had the examination. But I also got the results.

Guest B: Yes. In Norway, you take the test, but you do not get the results.

*[Several guests agreed.]*

Gynecologist: If everything is okay, you will not get notified about the results.

Guest B: For me, it is safer to see the results and be sure that nothing is wrong.

Guest C: For me, it is important to have a female doctor do the test. My GP is a man. And I often met him in church. It is awkward.

*[Some laughter from other guests.]*

Guest D: I know who you mean. We probably had the same doctor. But do you know you can change your GP on the internet? I have changed mine. It is easy.

This quote illustrates how several guests interacted and shared their personal experiences and opinions around gynecological examinations, including where to go for them, their reasons for not having them in Norway, or indeed what they experienced as a “safe” examination (e.g., more time for doctor–patient communication, access to results, and gender of the professional). The topic of gynecological examinations can be considered rather intimate in nature. However, this sharing of personal experiences also led to a discussion relating to the Norwegian health system, such as its practices around notifying patients of test results and how to change one’s GP. The quote also demonstrates that discussions and interactions occur not only between the health expert and guests but also among the guests. In contrast to the group participation and interactions in the organizational health parties, this discussion was not driven by the health expert, but by the extant and safe communications among the guests.

In Health Party 7, the same group of guests met the gynecologist. The topic of the meeting was menopause, which was chosen by the guests collectively. This time, not only did the guests share their personal experiences, but so did the gynecologist who had previously appeared rather reserved and acted in a strictly professional role. When the guests shared their symptoms of menopause and complained about their husbands’ lack of understanding, she offered her story:

Sometimes, we think men do not understand because they lack knowledge on menopause. But this is not always the case. I remember I was quite irritated by my husband. He is a doctor and knows the symptoms of menopause. But he forgot his wife can also have these symptoms. *[laughter from the others]*

This intervention is noteworthy because the gynecologist shifted between two participatory roles: the expert role as doctor and the social role as woman and wife. As the gynecologist made this shift, she joined in the group communication initiated by the guests. When the gynecologist brought her personal experience into the discussion, it became even clearer that different types of knowledge came into play, not only professional knowledge but also lay and experiential knowledge. A guest referred to this moment when speaking with the observing researcher during the break: “It was so interesting to hear ‘Hana’s’ [the gynecologist’s] experience with menopause troubles. I felt so relieved then, because you are not alone with these symptoms. I thought I was mad [referring to her unstable mood], you know.”

To summarize, the two ways in which health parties were organized resulted in markedly different participant roles and frameworks for communication.

Considering Goffman’s (1981) perspective on role play and participant frameworks, we may infer that the guests at organizational health parties were “assigned” more passive participant roles through certain formalities, such as how they were addressed in an organizational setting. As members of migrant organizations/communities, they were “summoned” to attend a meeting to listen and learn but not to share or speak. Interviewees described organizational health parties as resembling more a lecture or monologue than a “party” or other collective endeavor. By contrast, the guests at private health parties were “assigned” more active participant roles through both the private venue and the sharing of a meal that took place at these events.

Although the guests were assigned different participant roles based on the two ways of organizing a health party, the data also indicate that the participant role of the guest is a deliberate choice in the sense that it represents an individual choice to be active or passive. Such choices were connected to the type of social interactions that emerged in the health party. From a sociological perspective, individuals are involved in social structures and, as such, are governed by forces outside themselves. They are, however, also active agents (Bourdieu, 1980; Giddens, 1991; Goffman, 1974) and can choose to share and take part in discussions or to mainly participate as listeners. One of the interviewees said, “nobody *dares* to ask” when describing passive guest participation at an organizational health party. The word *dares* indicates a participant role formed by institutional or professional authority and power relations. It also suggests the importance of a sense of safety and security for active guest participation in health parties.

### The Role of Health Experts

One of NOOR’s intentions in establishing the health party program was to shine a light on issues of intercultural competence in the Norwegian health care system. Consequently, when creating the health expert team, they recruited several female health professionals with migrant backgrounds, including one gynecologist and several nurses. In this section, we will address the role of the expert in the interactions and discussions among guests at the health parties, particularly concerning their ethnic and cultural backgrounds. To illustrate this, we mainly use the example of the gynecologist because she participated as a health expert in several health parties. She was also the “expert” who received the most guest comments, as shown in the data analysis. According to the guests and hosts, the gynecologist showed a sensitivity toward the needs of guests who were migrant women and who wanted to learn more about gynecological topics. The results indicate that it could be important for the expert to have a migrant background, as she could better understand the meanings of the guests’ questions. Furthermore, she could respond not only as an expert but also as a migrant woman.

A Norwegian social worker, the host of Health Party 5, explained why she considered the gynecologist’s background to be beneficial in her role as health expert:

I think the health party is a smart way to inform migrant women. It is smart because the information is from a person who is from their own group. I feel that the message will reach them much better through people who are from the same country or share the same religious background than if it came through me. No matter how much knowledge I have on the topic, I just cannot approach them in the same way. Because I am Norwegian, I am white, while the health expert [referring to the gynecologist], at least she wears a hijab. They would then take the message more seriously and listen more. I think this is also because they know they would be understood. So, they would feel safer to ask questions if they do not understand.

This quote illustrates how difficult it could be for an expert to reach certain migrant participants with relevant and needed information. According to this interviewee, the migrant women responded differently to health information depending on who supplied it. She drew a contrast to herself with the gynecologist, who acted as the health expert in a health party she arranged and suggested that sharing a certain background with the group increased the guests’ receptivity to information.

Reflecting on the guests’ active engagement at the health party she organized, another interviewee, the host of Health Party 6, also emphasized the importance of the gynecologist’s identity:

Many of us are shy to talk in front of strangers. It is important that they feel safe to ask. “Hana” [the gynecologist] is such a pleasant person. I think they looked at her as one of us. Isn’t it? She also has an ethnic minority background. When she talked about the screening program, she said “When we moved to Norway, we were included in the Norwegian statistics.” So, we felt a kind of co-belonging when she spoke. Additionally, she is a woman. Many of us prefer female doctors, so she fits our group very well. That’s why the discussions went so well.

Although the interviewee referred to joint ethnic background that shaped a co-belonging or identification with a health expert, the gynecologist in fact shared neither ethnic nor religious background with the guests. Rather, she stressed the importance of migrant background and gender as a construction of a “sense of commonality.” This commonality caused guests and experts to identify with each other and influenced the interactions and participant roles. Both interviewees regarded the identity of the health expert as having an influence on the guests’ role as participant. The interviewee (host of Health Party 6) drew a contrast between the gynecologist and a stranger: “while a stranger may create an environment of uncertainty in which sharing is difficult, a person with a common background can create a sense of familiarity and, thus, security (*trygghet* in Norwegian) to speak and share.” Trust is implicated here, as trust is not only a way of managing uncertainty (Alaszewski & Coxon, 2009), but also one of the essential components of both health care communication (Chandra et al., 2018; McKinstry et al., 2006) and health care integration (Næss, 2019). As the interview data described, trust was shaped through joint identification, which was in turn based on intersecting categories of identity, such as ethnicity, migrant background, religion, and gender.

The interview with the gynecologist confirms such interpretation when she was asked whether her background mattered to her communications and interactions during the health party:

Yes, I think so. I have also experienced war and difficult life situations. So, my background, and that I have children myself, may have enabled me to understand how these women experience and feel . . . In addition, I know how the system works in this country after working in this system for many years now. This experience gives me insight and knowledge that I want to pass on to those who have just come, or who are in a situation where they don’t understand or have cultural distrust, or . . . things that have made it difficult for them to understand and accept. I think I understand that, and I want to contribute so that they get a better understanding.

Here, the gynecologist implied that her role as a health expert was influenced by personal experience as a migrant woman. As the health expert, she also underlined the significance of the expert capability to utilize cultural understanding in the health parties. She stressed how unfamiliarity with a situation and cultural differences with the expert may generate distrust and resistance to accepting certain aspects of Norwegian health care. In addition to talking about her migrant background and role as a mother, she also emphasized her knowledge of the Norwegian (health care) system through many years of experience working within it. This perspective corresponds with what Næss (2019) called “bridge-building” or “cultural health capital” when discussing the advantages of health care professionals with Somali backgrounds in their interactions with Somali migrants in Norway. In our analysis, the role of the expert and the type of understanding that was applied mattered. Not only were professional experience and expertise considered important, but also the capability to use one’s own cultural and personal experiences from war and the cultural distrust shared with participants. In this data analysis, we found that the role of the expert mattered, not in the sense that the expert and participants shared the same cultural or religious background, but because they have something in common, being migrants and being women, among other common experiences.

When reflecting upon how migrant background may influence the guests’ roles in the health parties, the gynecologist also underlined the importance of having a good reputation as health expert in a particular field and having a good informal relationship with migrant women:

Maybe some of the guests had met me before in the hospital, for example at birth or other occasions. When someone knows you, and experiences you as really nice and competent, it gives you a good reputation [such as]: “She is good, skilled, nice, or she is mean, unkind,” right? In addition, the host knows me. I think this created a sense of security among the guests, and they felt that they could trust me.

## Discussion

In the “Results” section, we describe different participant roles adopted during the health parties. Health experts are assigned a formal role in any health party, yet we see that the way the parties are organized matters, as does the way the expert role is interpreted and performed. They are expected to provide guests with correct information, answer questions, and act according to professional ethics and what they are usually paid to do. Their main task is to inform, but our results show that the role of the expert may also be to more actively engage participants with the issues addressed. The experts make it possible to relate these issues to one’s own personal experiences and life. In this sense, the role of the expert goes far beyond providing “expertise” or correct information about a health issue. The health party may present health experts with curious and engaged guests who dare to share lay knowledge and ask questions. In this matter, health parties provide activities for both health experts and participants to engage in and possibly gain a particular pedagogy (e.g., learn to interpret the description on the back of a bottle). At the health parties, roles other than the formal expert roles were communicated, particularly by the expert (gynecologist) who attended several health parties. She varied between the role of an expert and the role of a stranger to the Norwegian health care system, in addition to that of a migrant woman and mother. In the role of migrant woman, she shared her own experience with bodily changes related to aging and her interaction with a family member. When performing a variety of roles, including the personal role of migrant woman, the information she shared was probably not intimate, but it led to discussions beyond what is ordinarily expected in a formal and public doctor–patient relationship.

The participant roles of the hosts also differed. They all adopted a formal role as host when they introduced the topic and expert at the health parties and when they were the only one to ask questions following the expert’s presentation. In these situations, the host took a particular responsibility for facilitating the more active interaction between health experts and guests. The health party, thus, became a setting or arena for learning and knowledge acquisition. In the private setting of the health parties, such adoption of roles by a host led the guests to willingly ask questions to broaden their own knowledge and experiences during the second half of the party. This type of health party may demand less of the host, as the communication among the other participants and experts became a joint accomplishment. On the contrary, this kind of health party may demand more practical work from the host because these parties included meal preparation and service. The meal, and possibly also the informal talk following the first part of the health party, appeared to serve as a relaxing opening for participants. These health parties became a learning arena for discussing health issues and an arena for socializing. The hosts worked deliberately to develop the “social situation” and make it comfortable for the participants to open up and share knowledge and experiences. The guests’ participation and active engagement were influenced by how they interpreted the social situation and to what degree they “dared” to ask questions and talk. How they interpreted the social situation also influenced the trust between fellow guests and experts.

The question of trust seems crucial in this regard and is described as a central element in health communication and interaction (Chandra et al., 2018; Gabe et al., 2015; McKinstry et al., 2006). From a sociological perspective, trust is linked to risk and uncertainty (Giddens, 1991; Sztompka, 2000). Alaszewski and Coxon (2009) considered trust to be a way to manage or handle uncertainty, and the intention of the health party was to gather people who could trust each other to communicate and acquire knowledge about health issues, disease, and how to interact with the Norwegian health care system. Furthermore, Alaszewski and Coxon (2009) observed that trust is “embedded in personal relations and communications, so that when individuals encounter abstract and formal expert systems such as medicine they judge them in terms of the person who is the representative of the system” (p. 204). In other words, the person who acts as expert and disseminates information about health issues and health care systems may affect people’s level of trust in the health information disseminated and in the health care system. In Norway, health care and health care professionals are accustomed to a relatively high level of trust (Lian, 2003). However, a lack of institutional trust has been identified as a prevalent barrier to health care utilization by immigrants and ethnic minorities in not only Norway (Ayazi, 2006; Næss, 2019) but also other OECD countries (Feldmann et al., 2007; Manderson & Allotey, 2003; Vozikis & Siganou, 2015). A resonant illustration may be found in migrant women’s attitudes toward routine health screenings. Norwegian health care has an established screening system, including breast examinations and HPV screening, to detect and manage certain health risks (Norwegian Directorate of Health, 2019). However, these screenings must be accepted by the individual who has been offered the test, which in turn demands some trust in the medical system. Studies indicate that migrant women, particularly those who have lived in Norway for less than 10 years, do not attend these screenings as often as most of the population (Leinonen et al., 2017). As our data reveal, this situation has been addressed at a health party where the host encouraged the guests to change the situation and participate in the screening programs. It is a reason to add that ambivalence toward screening is not unique for the group we have studied, but found among different social groups and cultures for diverse reasons as stigma and misperceptions (Goldman et al., 2009), risk perceptions (Oster et al., 2013), and gender and class position (Hwang et al., 2017).

In particular, we use the health expertise provided by the gynecologist as an example. She illustrates how a health expert with migrant background may gain more positive access and trust from migrant women attending the health party. This is helpful as she could inform the women about how the Norwegian health care system works, what are the common diseases in this country, and how they are typically handled. Interviewees consider her participation in the health parties to be valuable because trust may be “embedded in personal relations.” By taking on the role of “participant,” she positively influenced migrant women by instilling trust in the health care system and by providing the information she shared with the guests.

The results also demonstrate how the two types of health parties (private vs. organizational) produced contrasting social settings that gave rise to different relationships among guests and possibilities to learn from each other’s experiences. This was not only a matter of social exchange but also a matter of reaching out to migrant women in a bottom-up manner. Collaborative interactions between experts, hosts, and guests are easily achieved in the private health party setting, yet dependent on the understanding of the expert role. Put differently, finding it useful to interact with the health care system and learn about health issues also depends on the pedagogy used by the health expert and on the framework applied for learning and sharing among migrant women.

### Limitations

The health party program analyzed in this study was conducted in only one county in Norway over a limited time period. Moreover, certain migrant communities, such as those from Eastern Europe, were not included in the study. It is, therefore, not clear how our results might be applied to a different or wider context. Nonetheless, such limitations are also potential strengths for qualitative studies like this, not least because such specificity provides thorough contextual knowledge and enables the deep and detailed thematic analysis that is essential for the improvement of health promotion in multicultural contexts.

### Implications

Based on the above findings, we argue that trust experienced by migrant women in the context of health education has relational (*whom* we listen to or share with) and situational (*when* and *where* we share) aspects. We conclude, therefore, that certain social interactions and social settings must exist if migrant women should deploy and develop trust and learn to interact with a public health care system. Furthermore, this should occur in the appropriate setting for active participation so migrant women can communicate their questions and be acknowledged for cultural differences compared with mainstream society and meet a health care system that adjusts to these circumstances. In this regard, certain informal aspects, such as creating a familiar and comfortable setting for interaction and securing the participation of key experts with whom participants can identify, are crucially important for establishing, maintaining, and increasing trust within migrant communities.
